# Cereal Processing By-Products as Rich Sources of Phenolic Compounds and Their Potential Bioactivities

**DOI:** 10.3390/nu13113934

**Published:** 2021-11-03

**Authors:** Anca Fărcaș, Georgiana Drețcanu, Teodora Daria Pop, Bianca Enaru, Sonia Socaci, Zorița Diaconeasa

**Affiliations:** Department of Food Science, Faculty of Food Science and Technology, University of Agricultural Science and Veterinary Medicine, 400372 Cluj-Napoca, Romania; anca.farcas@usamvcluj.ro (A.F.); georgiana.dretcanu@stud.ubbcluj.ro (G.D.); teodora-daria.pop@student.usamvcluj.ro (T.D.P.); bianca.enaru@stud.ubbcluj.ro (B.E.); sonia.socaci@usamvcluj.ro (S.S.)

**Keywords:** cereals, grains, by-product, brewers spent grain, polyphenols, bran, antioxidant, anticancer

## Abstract

In today’s society, we can see a progressive paradigm shift that tends towards a healthy and sustainable lifestyle. The proof is represented by the growing interest in food loss and waste of different sectors, from the political to the academic, or even to the private sector. In order to reduce food waste and to increase sustainability, the European Union (EU) has planned a circular bioeconomy. This action plan includes an approach based on reducing, reusing, recovering, and recycling materials and energy. Every year, there are high amounts of waste and by-products resulting from agricultural producing and agro-industrial processing, impacting the environment and the socio-economic sector. Cereal food products cover over 20% of daily diet, so it can be assumed that cereal production and processing are one of the most important sectors of agri-food industries. It is estimated that the waste generated from cereal processing and manufacturing is up to 13%, a percentage that can be decreased by converting the by-products in raw materials for biofuels, biodegradable plastics, alcohols, antioxidants, food additives, or pharmaceutic ingredients due to their content in macro- and micro-nutrients or bioactive compounds. Based on the fact that diet plays a crucial role in maintaining the integrity of our body, it is important to capitalize on any source of bioactive compounds to which we have access. This review aims to highlight the need to recirculate by-products for the purpose of extraction and use of their key compounds, polyphenols, which have not only antioxidant effects, but also preventive and therapeutic effects against cancer. For these, it is necessary to understand the biotechnologies needed for processing the most consumed cereals, the methods of extraction of phenolic compounds, and the main effects that these compounds have, summarizing the most relevant in vitro and in vivo studies performed so far.

## 1. Introduction

One of the biggest challenges for humanity is to live in a society without hunger but with high quality and safe food. In order to achieve this, we have to assume that worldwide food loss has to be drastically reduced [1]. Every year, approximately 1.3 billion tons of food loss and waste are generated from the whole food chain, including manufacturing, production, and consumption, representing close to one-third of the food produced [2]. Food loss and waste represent also a substantial consumption of important resources such as water, land, energy, and labor. These losses go hand-in-hand with the environmental deteriorations caused by the ineffective management of this waste. In order to reduce these insufficiencies, a viable solution is represented by the concept of circular bioeconomy, which have as prime goals the achievement of sustainability and economic viability for bio-stream production, a reduction in costs, the enhancement of competitiveness, and a reduction in poverty and hunger [3,4]. This paradigm shift from a linear towards a circular bioeconomy implies public awareness and acceptance, systems thinking, sustainable production and consumption, and, of course, zero discharge [5]. Some researchers associate this concept with the expression “waste-to-wealth”, aiming to convert renewable biological sources into high-end merchandise, to conserve the value of resources for a longer period with no generation of waste, and to reduce greenhouse gas emissions [3].

**Figure 1:**
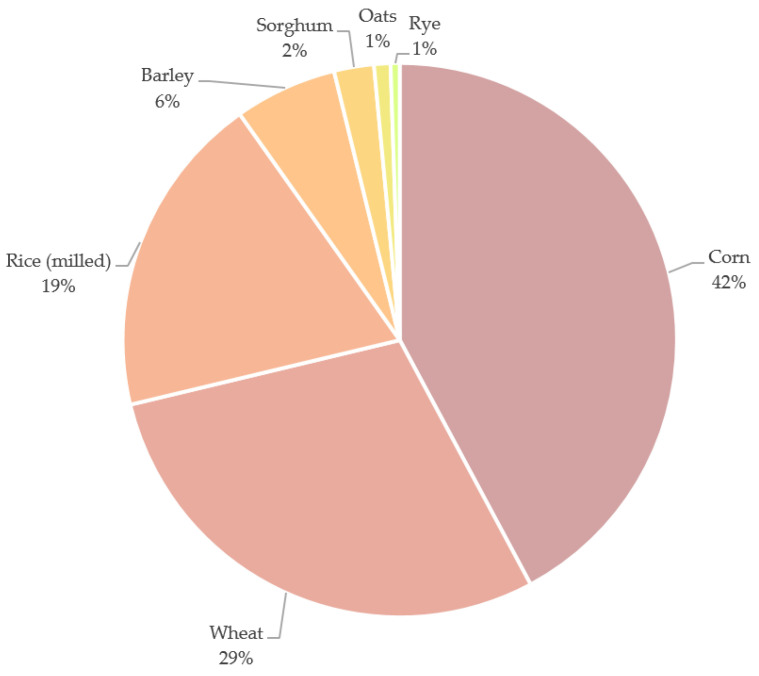
Percentage of worldwide cereals production during 2020/2021. Data source: Statista [8].

Along the food chain, high quantities of waste are generated during harvesting, transport, processing, storage, distribution and retailing, estimating a total amount of losses at 13% [6]. According to the Food and Agriculture Organization’s (FAO) last report from July 2021, the global cereal production reached 2817 million tons, whereas the production of coarse grains (grains other than rice and wheat used primarily for animal feed or brewing) reached 1513 million tons [7]. Cereals are members of the *Gramineae* family and are represented by nine species including wheat, rye, barley, oat, rice, millet, corn, sorghum, and triticale (a hybrid of wheat and rye). According to the reported data showed in Figure 1, corn, wheat, rice, and barley represent the majority of all the cereals produced worldwide in 2020/2021 (up to now) [8].

Knowing that cereal food products cover over 20% of daily diet, representing one of the most important industries from agri-food sectors, this paper intends to overcome with a complex overview of the by-products resulted from the most consumed cereals. Furthermore, this review will present not only the technological point of view related to most relevant sources of by-products in terms of cereals, but also their chemical composition, focusing on polyphenols, their extraction methods, and their potential health benefits, both in vitro and in vivo.

## 2. Cereal Processing

Processing techniques represent a group of operations that are performed in order to increase the starch availability and digestibility of the grains [9]. Moreover, studies have shown that processing methods are able to increase bioaccessibility and bioavailability of bioactive compounds and nutrients [10].

There are two types of conventional cereal processes—mechanical and chemical (which separate the wanted from the unwanted characteristics of the grain), followed by an enzymatic polishing process (which protects sensitive nutrients from thermal degradation and crack to which the cereals were previously subjected) [9]. Among all these conventional processes used in the cereal industry, the most common ones are dry milling applicable for wheat and rye, wet milling applicable for corn and sorghum, and pearling applicable for oat and barley (barley can also be processed by malting) [11].

### 2.1. Corn Processing

**Figure 2:**
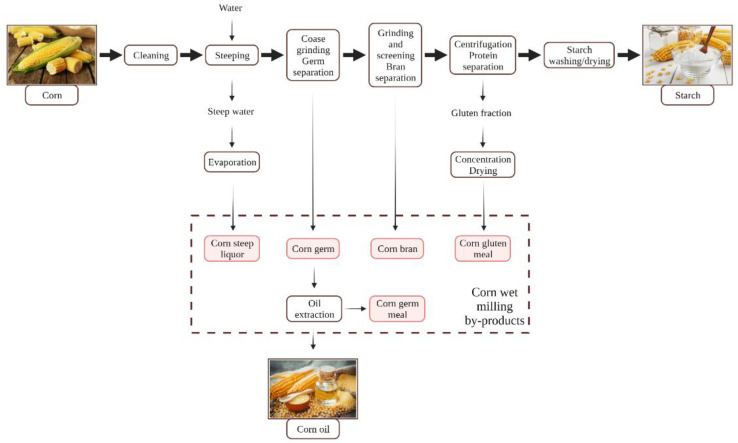
Types of by-products resulted after corn processing [11,17].

For corn, there are two milling techniques involved, including dry milling and wet milling. Our focus in this work is on wet milling, which is based on grain steeping in water and SO_2_ in order to soften the kernels and, in this way, to make the separation of the components easier. Wet milling has the aim of removing as many intact starch granules as possible, resulting in step solids, germ (for oil extraction), bran, and gluten as by-products. Starch (endosperm fractions) and oil (germ) are the major products of corn grain wet milling, while by-products include corn steep liquor, corn germ meal, corn bran, and corn gluten meal (Figure 2) [11]. Corn steep liquor consist of vitamins, minerals, and nitrogen (including amino nitrogen), which makes it a suitable material for fermentation [12].

Corn germ meal is a by-product resulted after the oil extraction from corn germ and contains a considerable amount of protein and essential amino acids including methionine (190 mg/100 g dry corn germ meal (dCGM)), cystine (210 mg/100 g dCGM), lysine (480 mg/100 g dCGM), arginine (720 mg/100 g dCGM), isoleucine (350 mg/100 g dCGM), leucine (830 g/100 g dCGM), valine (570 mg/100 g dCGM), and histidine (370 mg/100 g dCGM) [13]. Moreover, bioactive compounds such as tannins and carotenoids were found in considerable amounts in corn germ meal. Smuda et al. [14] revealed that tannins are found in 389.5 mg catechin equivalent (CE)/100 g, and carotenoids are found in 57.9 µg β-carotene/g in corn germ meal samples [14].

Compared with other cereals’ bran, corn bran is remarkable for the highest content of dietary fibers and phenolics. The insoluble dietary fibers are comprised of cellulose, hemicelluloses, and lignin fractions. Corn bran contains high quantities of protein, and some studies suggest that it contains more ferulic acid than other cereal brans (2.8–3.1 g/100 g corn bran), making corn bran a material with high antioxidant activity [11,15].

Corn gluten meal is one of the most important by-products from corn wet milling, resulting in a composition of approximately 60–70% crude protein [16]. Due to the imbalanced amino acid composition, corn gluten meal has a poor nutritional profile, which is why it is mostly marketed as feedstock or is discarded. However, when corn gluten meal suffers a hydrolysis treatment, it can produce peptides that are proven to have antioxidant activity [11].

### 2.2. Wheat Processing

**Figure 3:**
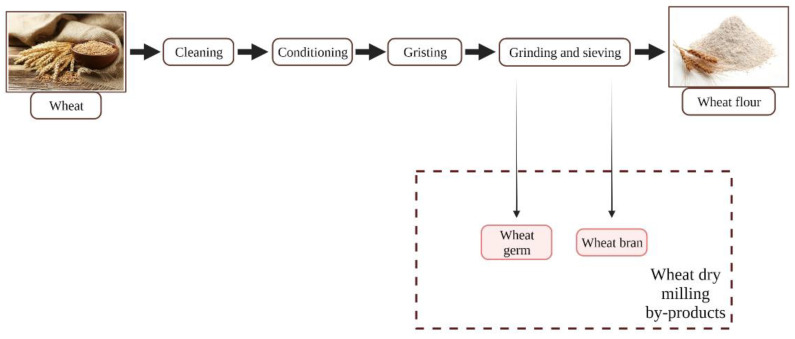
Types of by-products resulted after wheat processing [19].

Due to its unique rheological performance compared to other crops, wheat is the most important crop for the bread making industry, being the primary cereal produced in the EU [18]. When the final product is wheat flour for human consumption, the most common industrial processing is dry milling, which includes successive grinding and sieving operations, resulting in a good efficiency and high purity. During dry milling, bran and germs, as well as hulls and polish debris, are separated from the starchy endosperm. In the end, wheat bran, wheat feed, and germ represent the by-products, wheat bran being the most important one (Figure 3) [11].

Wheat bran is composed of the pericarp and the seed’s outermost tissues, including the aleurone layer, as well as varying quantities of residual starchy endosperm [18]. It contains dietary fiber as well as a wide range of biologically active compounds including alkylresorcinol (220–400 mg/100 g dry wheat bran (dWB)), ferulic acid (500–1500 mg/100 g dWB), β-glucan (2200–2700 mg/100 g dWB), and arabinoxylan (22,400–29,800 mg/100 g dWB) [15,20].

Wheat germ is considered not only a by-product due to its composition, such as lipids (10,000–15,000 mg/100 g dry wheat germ (dWG)), proteins (26,000–35,000 mg/100 g dWG), sugars (17,000 mg/100 g dWG), fiber (1500–4500 mg/100 g dWG), minerals (4000 mg/100 g dWG), as well as important quantities of bioactive compounds such as tocopherols (30–74 mg/100 g dWG), phytosterols (2.4–5 mg/100 g dWG), policosanols (1 mg/100 g dWG), carotenoids (0.4–3.8 mg/100 g dWG), thiamin (1.5–2.3 mg/100 g dWG), and riboflavin (0.6–1 mg/100 g dWG), but also a high valuable food supplement [19].

### 2.3. Rice Processing

**Figure 4:**
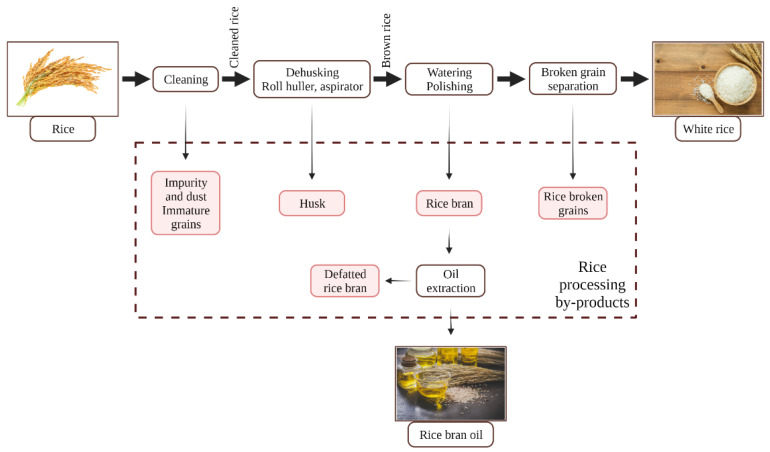
Types of by-products resulted after rice processing [11].

When talking about rice processing, there are important steps including harvesting as paddy rice, consisting of a thick, fibrous husk that firmly encases the grain, followed by milling, producing rice bran as a by-product. In order to achieve attractive white grains, rice is polished during processing. Paddy rice processing typically generates around 70% rice endosperm or white rice, which is the main output of rice production, followed by rice husk, rice bran, and rice germ as unconsumed rice fractions (Figure 4). The demand for rice production is expected to remain robust and stable over the next few decades, resulting in a growth of rice by-products. After processing, white rice grains primarily consist of carbohydrates, but they lack other critical elements. Therefore, rice by-products are more nutrient-dense than polished white rice [11].

Rice bran, which is a by-product of rice milling and accounts 10% of total paddy rice, is the most widely utilized feedstock, with the remainder being used for bran oil extraction. Rice bran is a mixture of outer layers and quantities of endosperm and germ produced during the brown-to-white rice milling process. Regarding its composition, rice bran contains significant amounts of proteins (11,000–15,000 mg/100 g dry rice bran (dRB)), carbohydrates (50,000 mg/100 g dRB), dietary fibers (11,500 mg/100 g dRB), oil (18–22 mL/100 g dRB), and a considerable amount of highly valuable bioactive phytochemicals, including phenolic compounds (157–665 mg gallic acid equivalents (GAE)/100 g dry rice bran), phytic acid (3500–5000 mg/100 g dRB), γ-oryzanol (152–912 mg/100 g dRB), α-tocopherol (4.13–4.61 mg/100 g dRB), γ-tocopherol (2.5–3.79 mg/100 g dRB), etc. [11,21,22]. Due to the high lipid content and the presence of lipases, rice bran has a limited shelf life, which is why it should be subjected to different heat-stabilization procedures. Its high oil content makes rice bran a useful raw material for rice bran oil production, producing the oil itself and a secondary by-product, defatted rice bran, which is usually disposed or used as a low-cost component for animal feed. Therefore, defatted rice bran contains high amounts of bioactive compounds such as phenolics, and it is a considerable source of protein [11].

Rice husk contains a considerable number of polyphenolic compounds, including silica and lignin, allowing husk to exhibit an antioxidant defense mechanism against oxidative stress [23].

Rice germ also contains a high crude protein content [18,000 mg/100 g dry rice germ (dRG)], as well as essential amino acids including arginine (1503.7 mg/100 g dRG), glutamic acid (1920.9 mg/100 g dRG), aspartic acid (1208.7 mg/100 g dRG), and leucine (1039.7 mg/100 g dRG), and significant quantities of fiber (7000 mg/100 g dRG). It is also abundant in water-soluble vitamins such as thiamine (B1) (5.69 mg/100 g dRG) and niacin (B3) (2.96 mg/100 g dRG), whereas vitamin E (11.96 mg/100 g dRG) is the major fat-soluble vitamin present in rice germ’s composition. Additionally, rice germ contains significant amounts of iron (77.5% of recommended dietary allowance (RDA)/100 g dRG) and magnesium (108% of RDA/100 g dRG), and due to its great nutritional value, rice germ presents a great potential, which certainly will be exploited in future studies [24,25].

### 2.4. Barley Processing in Brewing Industry

Barley is the fourth most cultivated cereal in the world, being mostly used as grain for animal feed and as malt for alcoholic beverages. The main processing mechanisms of barley grains are cleaning and dehulling, pearling, malting, and roller milling. Pearling is used to create polished grains; it is an abrasive process that gradually removes the seed coat, aleurone, sub aleurone layers, and the germ. During malting, grains go through a series of processes, such as steeping, germination, and kilning, the main by-products generated from these steps being malt sprouts and hulls [11]. The most significant quantity of malt by-products is further generated during the beer production. During mashing, the malted barley is mixed with water, and the temperature is slowly increased, promoting, in this way, enzymatic hydrolysis, when the starch is converted in fermentable and non-fermentable sugars, and proteins are partially degraded. After this step is produced a sweet liquid named wort. The wort is separated by the insoluble, undegraded, malted barley part, and the residual solid fraction is known as brewer’s spent grain (BSG) [26].

**Figure 5:**
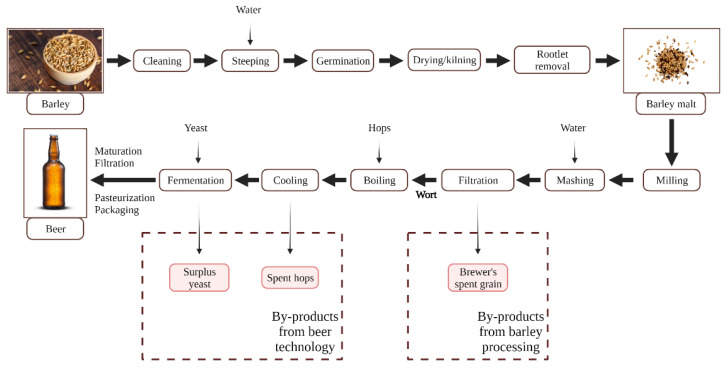
Types of by-products resulted after barley processing [32,33,34].

BSG is the major by-product produced in the brewing industry and is mostly made up of barley malt grain husks mixed with parts of the pericarp, seed coat layers, and fragments of the endosperm (Figure 5). Although it is generally known that BSG has a high concentration of carbohydrates, including fibers, proteins, lipids, and minerals and phenolic compounds, the chemical composition may vary. Some factors that can have significant influence include the botanical varieties of barley, the cultivation and harvest conditions, malting (light/dark roasted), and processing techniques. BSG’s essential amino acids contained includes lysine (14,300 mg/100 g dry BSG), leucine (6120 mg/100 g dry BSG), phenylalanine (4640 mg/100 g dry BSG), isoleucine (3310 mg/100 g dry BSG), and tryptophan (140 mg/100 g dry BSG). The non-essential amino acids from its composition are represented by histidine (26,270 mg/100 g dry BSG), glutamic acid (16,590 mg/100 g dry BSG), aspartic acid (4810 mg/100 g dry BSG), valine (4610 mg/100 g dry BSG), serine (3770 mg/100 g dry BSG), etc. BSG also contains a variety of minerals, the most prevalent of which are silicon (1074 mg/100 g dry BSG), phosphorus (518.6 mg/100 g dry BSG), and calcium (351.5 mg/100 g dry BSG) [27]. Numerous studies focus their attention on this by-product, because it represents a rich source of bioactive compounds, with phenolic compounds (1950–1620 mg GAE/100 g dry BSG), ferulic acid (156.53–290.89 mg/100 g dry BSG), syringic acids (12.22 mg/100 g dry BSG), catechin (8.44 mg/100 g dry BSG), gallic acid (3.22 mg/100 g dry BSG), and kaempferol (3.12 mg/100 g dry BSG) being some the most abundant ones from BSG’s composition [28,29,30,31].

## 3. Polyphenols in Cereal By-Products

**Figure 6:**
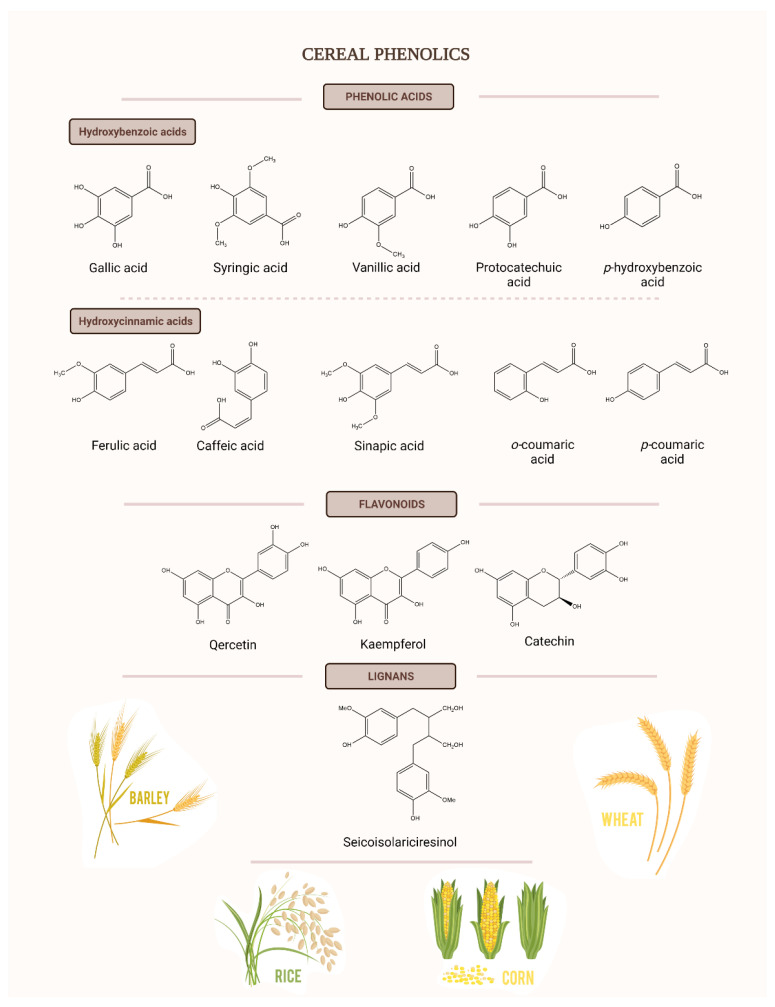
Principal phenolics in cereals (updated after Van Hung, P., 2016 [37]).

In the last decade, a significant number of studies have linked the cereal by-products, suggesting the presence in their matrix of a wide range of high value compounds, including polyphenols, which are secondary metabolites that are able to maintain and achieve an optimal cellular health. Additionally, polyphenols are among the largest classes of compounds with biological functions, in the case of humans, being intensively studied. In the case of cereals, the most common phenolic compounds are flavonoids and phenolic acids, which are classified as hydroxybenzoic and hydroxycinnamic acids (Figure 6) [35]. From the top produced grains all over the world, the phenolic content differs from one to another, corn being the one with the highest polyphenolic content (15.55 µg/g GAE/g of grain), followed by wheat (7.99 µg/g GAE/g of grain) and rice (5.56 µg/g GAE/g of grain). The content of polyphenols depends on a large number of factors, for example, brown rice contains more ferulic acid than the polished one because polyphenols are mainly found on the cortical layer. Even though polyphenols are distributed in all structural areas [36], the extracts analyzed from the bran always contained higher total phenolics compared to the endosperm (72.5–83.2% of total phenolic content is located in the outer fractions of the grain rather than the inner fractions) [37].

To obtain final products, cereal grains are usually milled, and during this process, the bran, which is separated from the starchy endosperm of the grain, represents a major by-product. Even though the nutrients are generally present in higher concentration in the outer part of the grain, it is often used as animal feed, being in this way undervalued. By-products contain significant amounts of bioactive compounds with specific health benefits, which can be extracted using several techniques, which will be presented further.

## 4. Extraction Methods

As mentioned before, phenolic compounds can be found in different layers of the grain, especially in bran, which represents the hard-outer layers of the cereal. That is the reason why this paper focuses on these by-products, because they are the major available sources of phenolics from the whole cereal grain. Thus, after the processing steps, where the bran is separated from the grain, an extraction step is required in order to benefit from the health-promoting potential of the targeted components [38].

In the last decade, researchers studied the influence of the physical and chemical processes used in the recovery of phenolic compounds and patented several protocols of the extraction methods. As shown in Table 1, there are different conventional and modern extraction methods that can be applied in order to target certain phenolic compounds/classes of phenolics. Besides the desired compounds, in order to choose the most sustainable method, there are several variables that should be taken into consideration, including time, yield, extract quality, cost, availability of the process, and environmental impact [39].

## 5. The Potential Health Effects of Extracted Polyphenols

### 5.1. Antioxidant Activity

**Figure 7:**
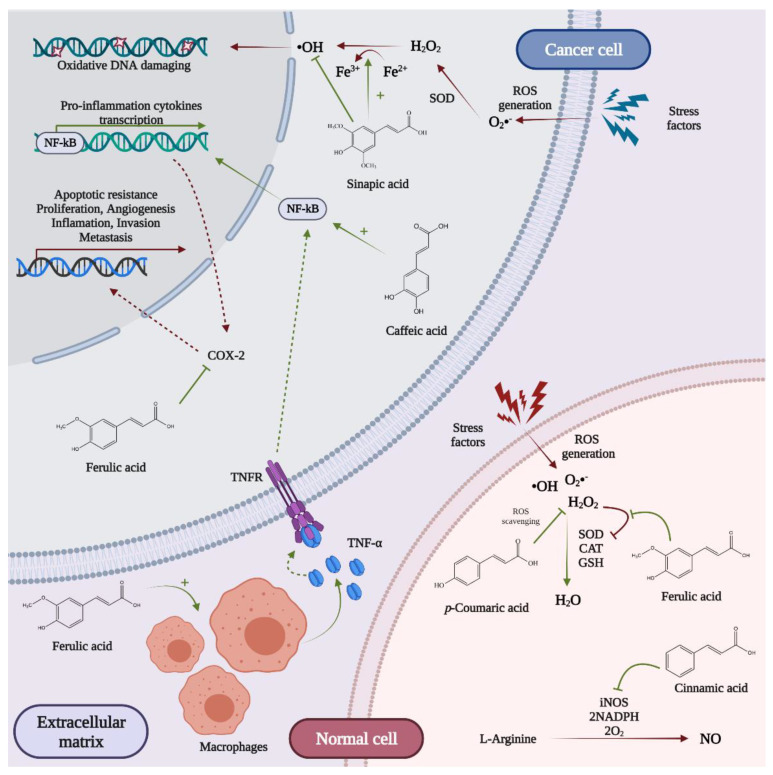
The main effects of phenolic compounds from cereal by-product extracts exert on normal and cancer cells.

The antioxidant system has the ability to regulate the generation and elimination of reactive oxygen species (ROS). Normally, mitochondria convert 1–2% of the consumed O_2_ to ROS, and under environmental stress, the ROS levels increase drastically [55]. A long-studied property of polyphenols is their antioxidant capacity [56]. This property is important when, at the cellular level, there is an imbalance between the production of ROS and/or reactive nitrogen species (RNS) and the defective antioxidant defense system [57]. Imbalance leads to oxidative stress, a cellular disorder that can cause many irreversible cellular damages (mutations). These damages can be linked to a series of pathogenesis such as cancer, diabetes, cardiovascular disease, Alzheimer’s disease, autism, aging, or neurodegenerative diseases [58]. After years of research, scientists found that phenolic compounds are able to inhibit ROS effects and protect macromolecules (such as proteins, lipids, and DNA) from oxidative degradation, possibly by iron ions chelation, preventing mutations, as showed in Figure 7 [43,59].

Therefore, as they have the ability to inhibit or prevent the oxidative damage, polyphenols are also known as antioxidant agents [60]. This is the main reason why, among cereal compounds, this paper focuses on polyphenols, due to their abilities to prevent and treat various types of diseases.

In this research field, sciences concentrate their efforts on compounds that can exhibit different mechanisms in order to achieve considerable results in both in vitro and in vivo studies, polyphenols representing the main phytochemicals that can impact normal and damaged cells, accessible not only in dietary sources, but also in by-products and wastes [61,62]. Many researchers, such as Martin-Diana et al. [63], have evaluated the possibility of incorporating polyphenols from by-products in food as nutraceutical ingredients in order to promote a circular bioeconomy and to enhance the nutritional and biological properties of daily meals. In her recent study, the team managed to evaluate the total antioxidant capacity of wheat and oat brans and discovered that bran polyphenols have a higher antioxidant capacity that whole grain polyphenols, according to DPPH assay. Even though both wheat and oat brans indicate good antioxidant activity, by comparing them, wheat bran polyphenols showed a better antioxidant capacity than oat bran, both according to ORAC assay (the antioxidant activity against peroxyl radicals) and to FRAP assay (the ferric reducing antioxidant power). In other words, at the cellular level, wheat bran polyphenols confer more efficient protection against lipid oxidation and hydroxyl radical formation and can reduce iron more effectively than oat bran polyphenols [63].

McCarthy et al. [64] emphasized in a paper that pale BSG phenolic extract protect the cells from H_2_O_2_-induced glutathione depletion, H_2_O_2_-induced decrease in superoxide dismutase (SOD) activity, and H_2_O_2_-induced decrease in catalase (CAT) activity. Additionally, they remarked that *p*-coumaric, ferulic, and cinnamic acids from corn bran extracts inhibited the production of nitric oxide (NO) by reduction in nitric oxide synthase (iNOS) expression in lipopolysaccharide (LPS)-induced RAW 264.7 macrophages [64]. Another novel relevant study performed by Guerrini et al. [65] evaluated the antioxidant activity of durum wheat bran and rice bran using two techniques—DPPH and ABTS radical scavenging assays. The results showed that, according to both tests, when the content of free phenolic molecules was equivalent, rice bran had a greater antioxidant activity than wheat bran. On the other hand, regarding bound phenolic extracts, durum wheat bran had a greater antioxidant activity than rice bran, both with DPPH and ABTS tests. It was also confirmed that the antioxidant capacity of cereal grains, in both in vitro and in vivo studies, is mainly due to the presence of the ferulic and phenolic acids in extracts’ composition [65]. Furthermore, there are data showing the possible antioxidant effects of wheat bran phenolics on humans due to their relatively good absorption rate [66].

### 5.2. Anti-Carcinogenic Effects

In recent decades, the molecular mechanisms behind the chemopreventive effects of polyphenols have been deepened in order to understand the effectiveness of these phytochemicals [67]. It is well known that polyphenols have the ability to manifest a series of properties, including the process of diminishing cell viability, inducing apoptosis, and modulating cell proliferation, inflammation, tumor growth, and metastasis [68]. In the following paragraphs will be outlined an overview of the main actions of phenolic extracts from by-products on various cancer lines.

#### 5.2.1. Cell Viability

Modulation of cell viability is a property can be exerted on both normal cells (only if the concentration of polyphenols is very high—this phenomenon is also called hormesis) [69] and cancer cells (in this case, the concentration can vary) [68].

In this context, Crowley et al. [70] managed to test different concentrations of polyphenols from two different sources—pale BSG and black BSG on two cancer cell lines, U937 and HepG2 [70]. For a little context, U937 is a pro-monocytic, human myeloid leukemia cell line [68], and HepG2 is a human liver cancer cell line [71]. The results of this group show that, on the U937 cell line, phenolic extract from black BSG have a high cytotoxic activity at low concentration (over 1%, *v/v*), while pale BSG extract failed to yield even at moderate concentrations (0–20%, *v/v*). On the other hand, in the HepG2 cell line case, both phenolic extracts did not have such a cytotoxic action comparable to the U937 line. The researchers also provided an explanation for these results—the HepG2 cell line has a number of metabolic enzymes with a detoxifying role. In other words, at the cellular level, metabolic enzymes would be able to rapidly inactivate harmful substances (e.g., polyphenols), giving this cell line increased resistance to BGS extracts [70].

Another interesting paper by Henderson et al. [59] highlights the impact of rice bran polyphenols on several cancer cell lines, such as colon (SW480 and HCEC cell lines) and breast (MDA-MB-468 and HBL100 cell lines). The phenolic extract consisted of tricin, ferulic acid, caffeic acid, and methoxycinnamic acid, and, in both cases, the phenolic extract managed to reduce the viability and decrease the colony-forming ability of the cells [59,72].

#### 5.2.2. Cell Proliferation and Apoptosis

Proliferation represents a crucial process in tumor development and progress, this being the result of the simultaneous stimulation of the signaling pathways of cell survival and growth [73]. For a compound to demonstrate its anticarcinogenic capacity, it must not only be able to stagnate proliferation, but it must also have pro-apoptotic properties in order to prevent tumor development and metastasis [74].

Tan et al. [75] presented that a mixture of phenolic extract from rice by-products is capable to inhibit cell proliferation in human colon adenocarcinoma cell line HT-29 [75,76]. Henderson et al. [59] obtained similar results, only that their extract from rice bran contained some specific polyphenols, such as ferulic acid, γ-oryzanol, phytic acid, coumaric acid, pectin, tricin, and tocotrienol-tocopherols [59].

Additionally, Kim et al. [77] tested three types of wheat bran extracts (mature, immature and steamed immature wheat bran extracts) for their anti-proliferative activity against Caco-2, HT-29, and HeLa cell lines, showing that, in all tested cells, all those three extracts inhibited the cell proliferation. After that, they tested the same extracts for the pro-apoptotic effects of phenolic molecules on the same cell lines and found out that immature wheat bran extracts increased the gene expression of p53 and PTEN (tumor suppressor genes) in HT-29 cell line, inhibiting the cell growth and inducing apoptosis [77].

#### 5.2.3. DNA Damaging

As mentioned previously, ROS and RNS can lead to high levels of oxidative stress and to high rates of DNA lesions, such as apurinic/apirimidinic sites (AP) generation, single or double-stranded breaks, or base substitutions. In normal cells, DNA damage response leads to the activation of reparatory mechanisms that can eliminate alterations, leading to a normal activity of the cell. On the other hand, in cancer cells, these mechanisms are inefficient, so the DNA can be easily damaged, due to the growing ROS levels, leading to cancer initiation and promotion [78].

In the tumor, polyphenols have double-effect in the case of ROS production; they can act as an antioxidant or as a pro-oxidant. Due to the presence of metal ions in the body (especially Cu^2+^), some polyphenols, which are generally effective antioxidants, can switch to a pro-oxidant behavior, which means that, in order to induce cell apoptosis via DNA breakage, they tend to induce ROS production exclusively in cancer cells [79]. From another perspective, polyphenols have the ability to decrease DNA damage induced by ROS accumulation, preventing, in this way, the evolution of the tumor [80]. The mechanism behind the genoprotective property of polyphenols consists of their ability to act as metal chelators in presence of ions, such as Cu^2+^ or Fe^2+^ [81].

This hypothesis is supported, for example, by McCarthy et al. [45], who demonstrated that the phenolic extract of BSG can protect the DNA from damaging by reducing reactive species and/or chelating ions in U937 cells. More specifically, the black BSG extract reduced the ferric oxidant Fe^2+^ to Fe^3+^, thus proving that it is a good genoprotective agent [45].

#### 5.2.4. Inflammation

Many cancers have some common molecular mechanisms of inflammatory process induction. One of them is based on upregulation of transcription factors, such as nuclear factor kappa-light-chain-enhancer of activated B cells (NF-kB). Studies showed that a significant number of phenolic compounds from cereal by-products (such as ferulic, caffeic, *p*-coumaric, and sinapic acids) are able to upregulate the NF-kB activity in a variety of cell lines, such as U9373xkB-LUC cells [81].

Another mechanism is the releasing of tumor necrosis factor alpha (TNFα), a mechanism described by Murakami et al. [82]. TNFα is a cytokine, a small molecule involved in cell signaling to induce inflammatory response [81]. Murakami et al. completed a study in which they found out that ferulic acid from BSG has a major anti-carcinogenic potential by releasing TNFα from RAW264.7 and a murine macrophage cell line (comparable to *p*-coumaric acid extracted from corn bran) and by suppressing the expression of cyclooxygenase-isoform 2 (COX-2) (comparable to caffeic acid extracted from BSG) [81,82].

## 6. Conclusions and Future Perspectives

It remains likely that cereal food products cover a large part of our daily diet, and their processing generates high amounts of waste and by-products. As today’s trend is to shift towards sustainability, there is a growth interest in reducing, recovering, and recycling the materials and energy. As cereal by-products consist of important phytochemicals, there is a focus on extracting and using those compounds in order to overcome certain humanity challenges. As presented above, polyphenols are a class of phytochemicals of interest due to their potential to exert biological functions, in the case of humans, by maintaining and achieving an optimal cellular health. There are several techniques for extracting the polyphenols from cereal by-products, but it is critical to use the most suitable one in order to benefits from its full potential. In vitro studies show the potential of polyphenols, both in normal and cancerous cells, by modulating the antioxidant defense system and the major signaling pathways responsible for cancer initiation. On the other hand, in vivo studies do not promote the effectiveness of these compounds due to their low bioavailability. In other words, when polyphenols are consumed as supplements or directly from dietary sources, they are rapidly metabolized in the upper digestive tract; thus, a lower concentration of the phenolic compounds reaches the cells. However, due to the new paradigm that focuses on the encapsulation and targeted administration of compounds, this limitation can be overcome, but it is necessary to carry out more studies regarding the targeted administration of polyphenols from by-products. Therefore, this work showed that cereal by-products are valuable sources of polyphenols, which are able to exert biological functions, a premise that can be effectively applied in the future, as the growing population requires an increasing cereal foods production, leading, in this way, to huge amounts of waste and by-products.

## Figures and Tables

**Table 1:** The main extraction methods of grains’ polyphenols.

Cereal By-Product	Extraction Method	Conditions	Polyphenols	References
Brewer’s spent grain	Supercritical carbon dioxide (SC-CO_2_)	- CO_2_ + Ethanol (0–60%, *v/v*) - *p* = 15–35 MPa - t° = 40–60 °C	Very low quantities of polyphenols	[2,40]
	Ultrasound Assisted Extraction (UAE)	- Acetone/Water (60/40%, *v/v*) or NaOH/Water (0.75/99.25%, *v/v*) - Ultrasound frequency = 20 kHz - t° = 39 → 47 °C	*p*-Hydroxybenzoic acid, Ferulic acid, Sinapic acid	[40,41]
	Microwave Assisted Extraction (MAE)	- NaOH (0.75%)/Choline chloride:glycerol - t° = 100 °C	*p*-Coumaric acid, Ferulic acid, Syringic acid	[40,42]
	Methanol/Methanol-Water	- V_Methanol_ = 10 mL - t° = 4 °C	Very low quantities of polyphenols	[40,43]
	Water	- V_H2O_ = 50 mL - t° = 25 °C; 80 °C	4-Hydroxybenzoic acid, *p*-Coumaric acid, Protocatechuic acid, Vanillin, Catechin, Vanillic acid	[44]
	Ethanol/ Ethanol-Water	- V_Ethanol_ (60–100%, *v/v*) = 50 mL - t° = 25 °C; 80 °C	4-Hydroxybenzoic, *p*-Coumaric acid, Protocatechuic acid, Vanillin, Catechin, Syringic acid	[44]
	Acetone/ Acetone-Water	- Acetone (60%, *v/v*) - t° = 60 °C	*p*-Hydroxybenzoic acid, Protocatechuic acid, Chlorogenic acid, 8-8′-DiFA, 5-5′-DiFA, 5-5′,8′-O-4′-TriFA, *p*-Coumaric acid, Ferulic acid, Sinapic acid	[41]
	H_2_SO_4_ and NaOH	- H_2_SO_4_ + NaOH - t° = 120 °C	Ferulic acid	[40]
	Saponification	- C_M_ = 1–4 M NaOH	Ferulic acid, *p*-Coumaric	[45]
Rice bran	Supercritical carbon dioxide (SC-CO_2_)	- CO_2_ + Ethanol (0, 5 and 10%) - t° = 40 °C, 60 °C - *p* = 30, 40 MPa	(+)-Catechin, Chlorogenic acid, Caffeic acid, *p*-Coumaric acid, Protocatechuic acid, Cyanidin-3-glucoside	[2,46]
	Ultrasound Assisted Extraction (UAE) for black and purple rice bran	- Solvent (20–60%) - t° = 30–60 °C - pH = 2–4		[2,46]
	Microwave Assisted Extraction (MAE)	- Methanol (100%) - t° = 185 °C - Microwave power = 1000 W		[46,47]
	Green method	- Glycerol (10–70%, *v/v*) - t° = 40–70 °C - Liquid-to-solid ratio = 10–40 mL/g		[2,46]
Corn bran	Pressurized alkaline hydrolysis	- C_M_ = 0.5 M NaOH—30% Ethanol - 15% Ammonia/Water - t° = 180 °C	Ferulic acid, *p*-Coumaric acid, Vanillin (derived)	[48]
	Single alkaline and acid extraction	- V_NaOH_ = 5 mL (2 N NaOH) - V_HCl_ = 5 mL (2 N HCl)	Vanillic acid, Cis-ferulic acid, *p*-Coumaric acid, Caffeic acid, Syringic acid, Sinapic acid	[49]
	Single neutral extraction	- V_Ethanol_ (50%, *v/v*) = 10 mL		[49]
	Acetone	- V_Acetone_ (50%, *v/v*) = 20 mL - t° = 25 °C	*p*-Coumaric acid, Syringic acid, Ferulic acid	[50]
Wheat bran	Supercritical carbon dioxide (SC-CO_2_)	- CO_2_ (8 ± 1 kg CO_2_/h) - t° = 40 ± 2 °C - *p* = 25.0 ± 1.0 MPa	*p*-Hydroxybenzaldehyde, Ferulic acid, Syringic acid, Syringic aldehyde, Vanillic acid, Vanillin	[51]
	Ultrasound Assisted Extraction (UAE)	- Methanol/Ethanol/Acetone (70/70/70%, *v/v*) - Ultrasound frequency = 40 kHz - t° = 50 °C		[52]
	Steam Explosion	-Ethanol -t° = 224 °C -*p* = 2.5 MPa	Ferulic acid predominantly	[53]
	Enzymatic Hydrolysis	- Multi-enzyme complex Viscozyme L/Xylanase/Feruloyl esterase	Ferulic acid	[54]

## Abbreviations

EU	European Union
FAO	Food and Agriculture Organization
dCGM	Dry Corn Germ Meal
CE	Catechin Equivalent
dWB	Dry Wheat Bran
dWG	Dry Wheat Germ
dRB	Dry Rice Bran
GAE	Gallic Acid Equivalent
dRG	Dry Rice Germ
RDA	Recommended Dietary Allowance
BSG	Brewer’s spent Grain
SC-CO_2_	Supercritical Carbon Dioxide
UAE	Ultrasound Assisted Extraction
MAE	Microwave Assisted Extraction
ROS	Reactive Oxygen Species
RNS	Reactive Nitrogen Species
CAT	Catalase
NO	Nitric Oxide
iNOS	Nitric Oxide Synthase
LPS	Lipopolysaccharide
AP	Apurinic/Apirimidinic
NF-kB	Nuclear Factor Kappa-light-chain-enhancer of activated B cells
TNFα	Tumor Necrosis Factor alpha
COX-2	Cyclooxygenase-isoform 2
